# Effects of Vitamin K_2_ on the Expression of Genes Involved in Bile Acid Synthesis and Glucose Homeostasis in Mice with Humanized PXR

**DOI:** 10.3390/nu10080982

**Published:** 2018-07-27

**Authors:** Halima Sultana, Kimika Watanabe, Md Masud Rana, Rie Takashima, Ai Ohashi, Michio Komai, Hitoshi Shirakawa

**Affiliations:** Laboratory of Nutrition, Graduate School of Agricultural Science, Tohoku University, 468-1 Aramaki Aza Aoba, Aoba-ku, Sendai 980-8572, Japan; halima@g-mail.tohoku-university.jp (H.Su.); k.watanabe@g-mail.tohoku-university.jp (K.W.); masudrana@g-mail.tohoku-university.jp (M.M.R.); takashima.rie@opal.plala.or.jp (R.T.); happy-go-luckygirl.ai.221@docomo.ne.jp (A.O.); mkomai@m.tohoku.ac.jp (M.K.)

**Keywords:** PXR, menaquinone 4, bile homeostasis, energy homeostasis, gene expression

## Abstract

Pregnane X receptor (PXR) is a nuclear receptor activated by various compounds, including prescribed drugs and dietary ingredients. Ligand-specific activation of PXR alters drug metabolism and affects many other physiological conditions. Species-specific ligand preference is a considerable challenge for studies of PXR function. To increase translational value of the results of mouse studies, humanized mouse model expressing human PXR (hPXR) has been developed. Menaquinone-4 (MK-4), one of vitamin K_2_ analogs prescribed in osteoporosis, is a PXR ligand. We hypothesized that MK-4 could modulate the physiological conditions endogenously influenced by PXR, including those that have not been yet properly elucidated. In the present study, we investigated the effects of a single oral treatment with MK-4 on hepatic gene expression in wild-type and hPXR mice by using quantitative RT-PCR and DNA microarray. MK-4 administration altered mRNA levels of genes involved in drug metabolism (*Abca3*, *Cyp2s1*, *Sult1b1*), bile acid synthesis (*Cyp7a1*, *Cyp8b1*), and energy homeostasis (*Aldoc*, *Slc2a5*). Similar mRNA changes of *CYP7A1* and *CYP8B1* were observed in human hepatocarcinoma HepG2 cells treated with MK-4. These results suggest that MK-4 may modulate bile acid synthesis. To our knowledge, this is the first report showing the effect of MK-4 in hPXR mice.

## 1. Introduction

The pregnane X receptor (PXR, also known as SXR or NR1I2) was identified by three different research groups in 1998 as a member of the nuclear receptor superfamily of ligand activated transcription factors. PXR is mainly expressed in the liver and intestine—the major organs where detoxification occurs [1,2,3]. This receptor is generally known to be activated by xenobiotics and pharmacological compounds; it plays an important role in the activation of many drug metabolizing enzymes and membrane-bound drug transporters. Among these enzymes, the most important are those of the 3A (CYP3A) subfamily of the cytochrome P450 superfamily because they are involved in the metabolism of approximately 50% of prescribed drugs [4,5].

Nuclear receptors have common structural features: an N-terminal domain, a DNA-binding domain (DBD), and a ligand binding domain (LBD). PXR has attracted particular attention because of its enlarged, flexible, hydrophobic LBD that allows PXR to be activated by a large variety of substances. Because of its structural features, PXR LBD can change its shape, thereby fitting various ligands, depending on their nature [6]. PXR can be activated by xenobiotic compounds, pharmacological agents, dietary substances, and endogenous lithocholic acid [6,7,8,9,10]. Moreover, recent studies have revealed the involvement of PXR in many physiological functions other than drug metabolism, such as inflammation, bile synthesis, bone homeostasis, vitamin D metabolism, lipid homeostasis, energy homeostasis, and cancer [9].

Vitamin K (VK) is a well-known nutrient that acts as a co-factor of γ-glutamyl carboxylase and plays an important role in blood coagulation and bone formation. VK is also involved in many physiological and pathological conditions, such as inflammation, testosterone production, cancer progression, and type 2 diabetes [11,12,13,14,15]. Inadequate intake of VK may be associated with osteoporosis, oseteoarthritis, Alzheimer’s disease, and coronary artery disease [16,17,18]. Menaquinone-4 (MK-4) is a vitamin K_2_ analog, which has been used as a therapeutic agent in osteoporosis in many East and South East Asia countries [19].

In 2003, another mode of action of MK-4 has been revealed by Tabb et al. [14]. It was demonstrated that MK-4 directly binds to and transcriptionally activates PXR as well as promotes the association between PXR and coactivators. Activated PXR, in turn, plays an important role in bone homeostasis by modulating gene expression [14]. The function of PXR activation by MK-4 in bone homeostasis was further evaluated by analyzing gene expression changes caused by both rifampicin (Rif) and MK-4 [20]. Later, PXR-mediated effect of VK was also shown in human hepatocellular carcinoma [21]. However, the research related to the PXR-mediated effects of MK-4 has concentrated mostly on bone physiology and cell-based experiments. Therefore, potential effects of MK-4 mediated by the activation of PXR should be investigated in other organs. This may shed light on many mechanisms related to diseases in which VK can be used as a preventive or therapeutic agent.

Species-specific ligand preference by PXR constitutes a significant challenge for the studies of PXR function. For example, pregnane 16α-carbonitrile (PCN) is a much stronger activator of rat or mouse PXR than of human or rabbit PXR. Similarly, Rif is a strong activator of human or rabbit PXR but a very weak activator of mouse or rat PXR [22]. These species-specific preferences show that evaluation of toxicity and functionality of PXR ligands in rodents has an inherent limitation as it may not be readily translated into human physiology. To overcome this issue, several groups of researchers have developed a mouse model with humanized PXR by using different strategies [4,23,24,25].

In the present study, we used the humanized mouse line (hPXR) in which the LBD region of the human *PXR* gene was homologously knocked-in to the mouse *Pxr* gene, replacing the sequence that encoded endogenous LBD [4]. We then measured changes in mRNA expression levels after oral administration of MK-4 in hPXR and wild-type (WT) mice. We found that MK-4 affected expression levels of genes involved in bile acid synthesis and energy homeostasis mostly in hPXR rather than in WT mice. Lastly, we evaluated the effect of MK-4 on the expression levels of bile acid synthesis genes in human hepatocarcinoma HepG2 cells.

## 2. Materials and Methods

### 2.1. Materials

MK-4 was kindly provided by Nisshin Pharma Inc. (Tokyo, Japan). Dimethyl sulfoxide (DMSO) and Rif were purchased from Wako Pure Chemicals (Osaka, Japan). Corn oil (J-Oil Millis, Inc., Tokyo, Japan) was purchased just before every experiment from the local grocery.

### 2.2. Animals

The experimental plan for this study was approved by the Animal Research and Animal Care Committee of the Tohoku University (2014Noukumikae-026, 2017Noukumikae-006). All experiments were conducted under the guidelines issued by this Committee in accordance with Japanese governmental legislation (2005) that establishes the rules for the care and use of animals in animal studies.

hPXR mice on the C57BL/6NCrSlc background, originally established by Igarashi et al. [4], were obtained from RIKEN BioResource Center (Tsukuba, Japan). Homozygous hPXR and WT female mice (12–13 weeks of age) were maintained in plastic cages (3–4 mice per cage) with free access to commercial diet (CRF-1, Oriental Yeast Co. Ltd., Tokyo, Japan) and tap water under controlled temperature (23 ± 2 °C), humidity (50 ± 10%) and a 12:12 light:dark cycle (lights on at 8:00). To reduce the dietary interference, after 14–16 h of fasting, MK-4 at a concentration of 10, 50, or 100 mg/kg body weight (BW) or 50 mg/kg BW Rif dissolved in corn oil containing 0.1% DMSO was given to the mice by oral gavage. Control mice received only the vehicle. In 6 h after gavage, the mice were sacrificed and a part of the liver was preserved immediately in RNAlater (Thermo Fishier Scientific, Waltham, MA, USA). The remaining part was snap-frozen in liquid nitrogen and then preserved at −80 °C for further analysis. We have decided this experimental duration with reference to hepatic MK-4 contents after the treatment with MK-4 in wild type mice (data not shown).

### 2.3. Cell Culture

Human hepatocarcinoma HepG2 cells were maintained in Dulbecco’s modified Eagle’s medium (DMEM; Sigma-Aldrich Co., St. Louis, MI, USA) supplemented with 10% fetal bovine serum and antibiotics (100 U/mL penicillin and 100 μg/mL streptomycin; Gibco, Thermo Fisher Scientific) at 37 °C in a humidified atmosphere of 95% air and 5% CO_2_. Cells were seeded into 35 mm plates at a density of 2.5 × 10^5^ cells per plate and incubated overnight. The culture medium was then replaced with fresh medium, and MK-4 was added to the cells at a final concentration of 0 (control), 10, 20, or 30 μM.

### 2.4. High-Performance Liquid Chromatography (HPLC) for Determination of MK-4 Levels in the Liver

Liver samples (0.1 g) were homogenized in five volumes of 66% 2-propanol. VK was extracted from the homogenate using six volumes of *n*-hexane as described previously [26] and measured using a fluorescent HPLC system (Agilent 1260 infinity, Agilent Technologies, Santa Clara, CA, USA; Puresil C18, 5 µm, 4.6 × 150 mm column; Waters Co., Milford, MA, USA; RC-10, 4.0 × 50 mm, Shiseido-IRICA, Kyoto, Japan; fluorescence detection, excitation at 240 nm, emission at 430 nm). The concentration of MK-4 was calculated using the relative fluorescent intensities of menaquinone-3 (Eisai Co., Ltd., Tokyo, Japan) as internal standard [26].

### 2.5. RNA Extraction and mRNA Quantification

Total RNA was isolated from the liver and HepG2 cells using the Isogen reagent (Nippon Gene, Tokyo, Japan) according to the manufacturer’s instructions. The quantity and quality of RNA were determined spectrophotometrically, by measuring the absorbance at 260 nm in relation to that at 280 nm, and subsequent agarose gel electrophoresis. Four microgram of total RNA was denatured at 65 °C for 5 min with 2.5 μM oligo-dT primer (Hokkaido System Science Co., Sapporo, Japan) and 0.5 mM dNTP (GE Healthcare, Tokyo, Japan) for cDNA synthesis. RNA was incubated in 20 μL of RT buffer (50 mM Tris–HCl (pH 8.3), 75 mM KCl, 3 mM MgCl_2_, 5 mM dithiothreitol) containing 50 U SuperScript III reverse transcriptase (Invitrogen, Carlsbad, CA, USA) and 20 U RNaseOUT RNase inhibitor (Invitrogen) for 60 min at 50 °C. An aliquot of synthesized cDNA was used as template for quantitative RT-PCR (qRT-PCR) using an Applied Biosystems 7300 Real-Time PCR System (Foster City, CA, USA). Target cDNAs were amplified using gene-specific primers (Table 1) and SYBR Premix Ex Taq (TaKaRa Bio, Otsu, Japan) solution. The relative mRNA levels were normalized to the amount of eukaryotic translation elongation factor 1α1 (*Eef1a1*) mRNA [27].

### 2.6. DNA Microarray

Pooled RNA of each group (100 µg) was submitted to TaKaRa Bio (Otsu, Japan), where whole gene expression was analyzed by using Agilent mouse DNA array (SurePrint G3 Mouse GE v2 8x60K). Expression levels of genes upregulated (more than twice) or downregulated (to below 0.5-fold) by MK-4 and Rif treatments were compared to those in control group. Selected genes were analyzed by the web-based software David (https://david.ncifcrf.gov/) and Enricher (http://amp.pharm.mssm.edu/Enrichr/).

### 2.7. Statistical Analysis

Data are presented as the mean  ±  SEM (standard error of mean). Statistical analysis was performed by the Student’s *t*-test or Dunnet’s test using SigmaPlot version 12.5 (San Jose, CA, USA). All statistical analyses were conducted with a significance level of α = 0.05 (*p* < 0.05).

## 3. Results

### 3.1. MK-4 Concentration in the Liver of hPXR and WT Mice

**Figure 1:**
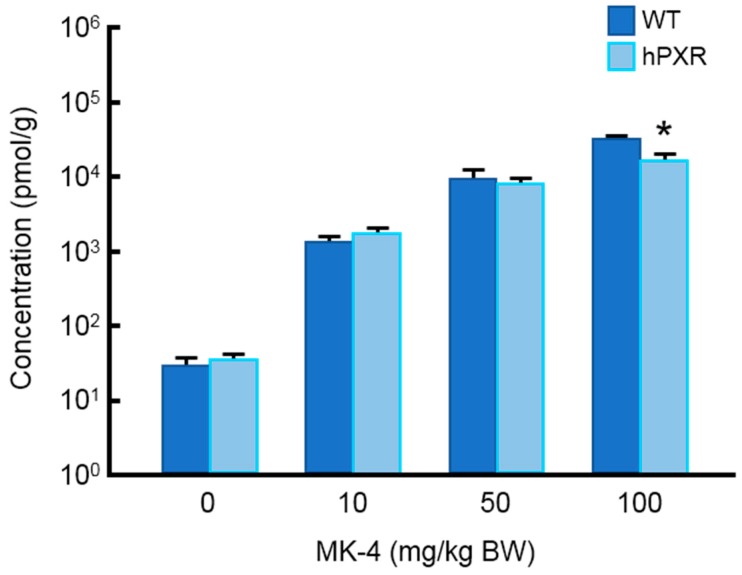
Menaquinone-4 concentration in the liver of wild-type and hPXR mice after treatment with menaquinone-4. Menaquinone-4 (10, 50, or 100 mg/kg of body weight) was given by oral gavage to wild-type (WT) and hPXR female mice. After 6 h, mice were sacrificed, and hepatic menaqinone-4 concentrations were quantitated by fluorescence HPLC. Data are expressed as the mean ± SEM, *n* = 4–9. * *p* < 0.05, compared to wild-type groups.

The body weight, liver weight, and liver/body weight ratio of WT and hPXR mice treated by MK-4 are shown in Table 2. There was no significant difference among the groups. We determined MK-4 concentration in the liver by fluorescence HPLC. MK-4 content was elevated in a dose-dependent manner, but no significant difference was observed between MK-4 levels in hPXR and WT mice except for when the highest dose of MK-4 was used (Figure 1).

### 3.2. Effects of MK-4 and Rif on mRNA Levels of Typical PXR Target Genes

**Figure 2:**
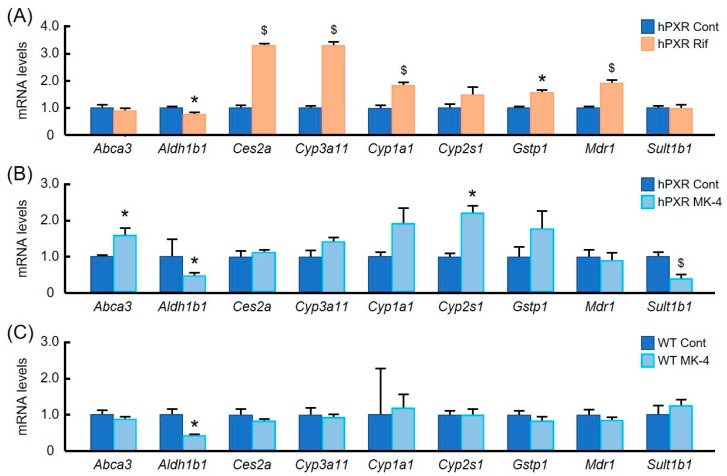
Effects of menaquinone-4 and rifampicin on the expression of genes encoding drug metabolizing proteins in the liver of wild-type and hPXR mice. Rifampicin (Rif; 50 mg/kg of body weight) was given by oral gavage to hPXR female mice (**A**). Menaquinone-4 (MK-4; 100 mg/kg of body weight) was given by oral gavage to hPXR (**B**) and wild-type (**C**) female mice. After 6 h, mRNA levels of genes that encode drug metabolizing proteins were measured by quantitative RT-PCR. The relative mRNA levels were normalized to the amount of eukaryotic translation elongation factor 1α1 mRNA. Data are expressed as the mean ± SEM, *n* = 4–5. ^$^ *p* < 0.01, * *p* < 0.05, compared to control groups.

First, by using qRT-PCR, we analyzed mRNA levels of some well-known PXR target genes and of some other genes involved in drug metabolism in the liver after the treatment of hPXR mice with Rif or MK-4 (Figure 2). mRNA levels of typical PXR target genes—such as carboxy esterase 2a (*Ces2a*), cytochrome P-450 3a11 (*Cyp3a11*), glutathione S-transferase pi 1 (*Gstp1*), and multi drug resistance 1 (*Mdr1*)—were markedly upregulated by the treatment with Rif (Figure 2A) in hPXR mice. However, MK-4 treatment had no effect on the expression of any of these genes (Figure 2B) in hPXR mice. MK-4 treatment significantly affected mRNA levels of ATP-binding cassette, sub-family A, member 3 (*Abca3*), cytochrome P450, family 2, subfamily s, polypeptide 1 (*Cyp2s1*), and sulfotransferase family 1B, member 1 (*Sult1b1*) genes (Figure 2B), which are not known as common targets of PXR. In contrast, WT mice were almost unaffected by MK-4 treatment (Figure 2C).

### 3.3. MK-4 and Rif Induced Different Changes in Gene Expression in hPXR and WT Mice

**Figure 3:**
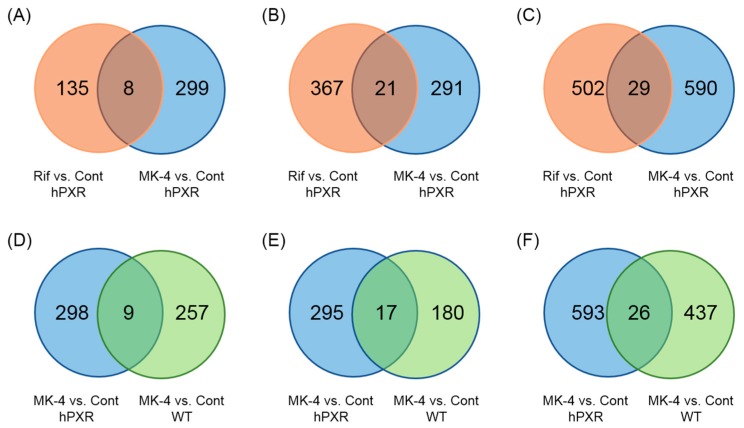
Venn diagram of differentially expressed genes in mice treated with rifampicin, menaquinone-4, and control mice. (**A**–**C**) Genes that were (**A**) upregulated (more than twice), (**B**) downregulated (to below 0.5-fold), or (**C**) overall differentially expressed after the treatment with rifampicin (Rif) or menaquinone-4 (MK-4) in comparison with the levels in control (Cont) hPXR mice. Overall, there were 29 shared differentially expressed genes between Rif- and MK-4 treated groups (eight upregulated and 21 downregulated). Five hundred and two genes (135 upregulated and 367 downregulated) and 590 genes (299 upregulated and 291 downregulated) differentially expressed genes were unique for Rif- and MK-4 treated hPXR mice, respectively. (**D**–**F**) Genes that were (**D**) upregulated (more than twice), (**E**) downregulated (to below 0.5-fold), or (**F**) overall differentially expressed in comparison with the levels in control (Cont) WT and hPXR mice. Overall, there were 26 shared differentially expressed genes between MK-4 treated hPXR and WT groups (nine upregulated and 17 downregulated). Five hundred and ninety-three (298 upregulated and 295 downregulated) and 437 (257 upregulated and 180 downregulated) differentially expressed genes were unique for MK-4 treated hPXR and WT mice, respectively.

To establish whether genes other than those involved in drug metabolism are affected by the treatment with MK-4, the DNA microarray assay was performed. From DNA microarray gene expression analysis of pooled RNA, we found that the effects of MK-4 on PXR target genes in hPXR mice were different from those of Rif or MK-4 in WT mice (Figure 3). Selected genes, whose expression was upregulated (to more than twice) or downregulated (to below 0.5-fold) in MK-4 and Rif treated groups in comparison with that in control, were analyzed by web-based software programs, David and Enricher. We found that some genes involved in bile acid synthesis and energy homeostasis were affected by MK-4 treatment in hPXR mice. Expression levels of these genes in MK-4-treated hPXR and WT mice as well as in Rif-treated hPXR mice are compared in Table 3. The ratios were deduced by comparisons with respective control groups. From DNA microarray data, we did not find any alteration in the expression of genes related to liver injury, indicating that up to the highest dose (100 mg/kg BW) of MK-4 administered in this study could not cause liver damage.

### 3.4. Validation of the Data Obtained from DNA Microarray Analysis by Quantitative RT-PCR

**Figure 4:**
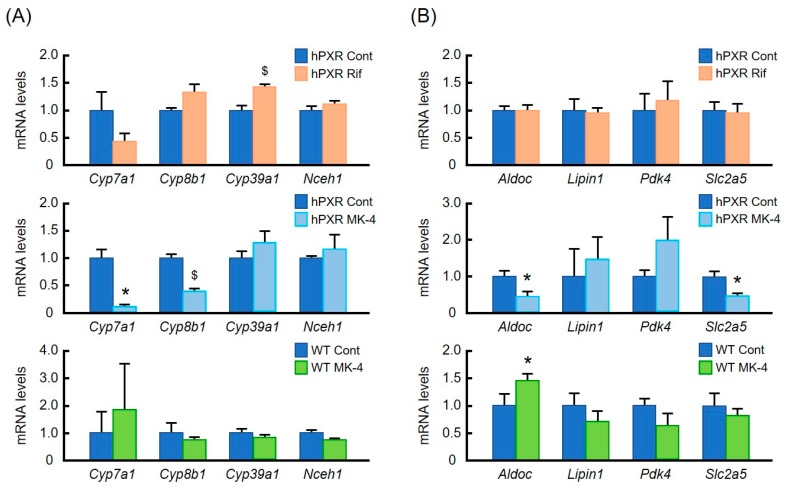
Effects of menaquinone-4 and rifampicin on mRNA levels of genes involved in bile acid synthesis and energy homeostasis in the liver of wild-type and hPXR mice. Menaquinone-4 (MK-4; 100 mg/kg BW) was given by oral gavage to wild-type (WT) and hPXR female mice. Rifampicin (Rif; 50 mg/kg BW) was given by oral gavage to hPXR female mice. After 6 h, mRNA levels of genes regulating bile acid synthesis (**A**) and energy homeostasis (**B**) were measured by quantitative RT-PCR. The relative mRNA levels were normalized to the amount of eukaryotic translation elongation factor 1α1 mRNA. Data are expressed as the mean ± SEM, *n* = 4–5. ^$^ *p* < 0.01, * *p* < 0.05, compared to control groups.

Given that the DNA microarray assay was done using pooled RNA from each group, DNA microarray data on the expression of bile acid synthesis and energy homeostasis genes were further validated by qRT-PCR. We found that gene expression data obtained from qRT-PCR were comparable with DNA microarray data (Table 3 and Figure 4). In accordance with other studies, Rif had a tendency to suppress *Cyp7a1* (cytochrome P450, family 7, subfamily a, polypeptide 1) expression in hPXR mice although the effect did not reach statistical significance. *Cyp8b1* mRNA level (cytochrome P450, family 8, subfamily b, polypeptide 1) was not significantly altered by Rif treatment either (Figure 4A, Upper). However, mRNA levels of these genes were significantly reduced by MK-4 treatment in hPXR mice (Figure 4A, Middle), whereas in WT mice, expression levels of these genes were not significantly altered (Figure 4A, Lower). Furthermore, although none of the energy homeostasis genes were affected by Rif treatment, expression levels of *Aldoc* (aldolase C) and *Slc2a5* (solute carrier family 2 member 5) were significantly suppressed by MK-4 treatment in hPXR mice (Figure 4B, Upper and Middle). Interestingly, MK-4 treatment increased mRNA level of *Aldoc* in WT mice. However, expression levels of other energy homeostasis genes were not affected in WT mice (Figure 4B, Lower).

### 3.5. Effects of Lower Doses of MK-4 on Gene Expression

**Figure 5:**
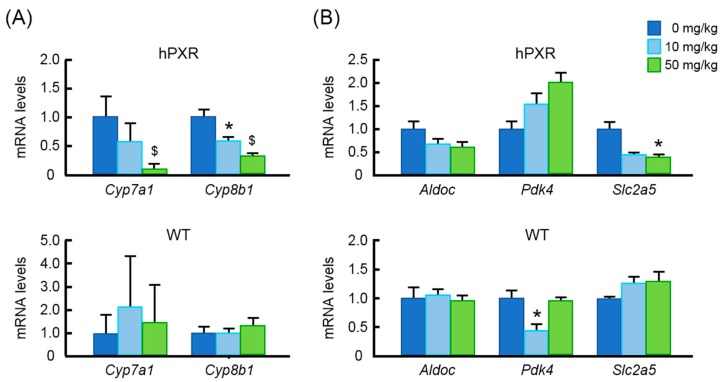
Effects of lower doses of menaquinone-4 on the expression of genes involved in bile acid synthesis and energy homeostasis. Menaquinone-4 (MK-4; 10 or 50 mg/kg BW) was given by oral gavage to wild-type (WT) and hPXR female mice. After 6 h, mRNA levels of genes regulating bile acid synthesis (**A**) and energy homeostasis (**B**) were measured by quantitative RT-PCR. The relative mRNA levels were normalized to the amount of eukaryotic translation elongation factor 1α1 mRNA. Data are expressed as the mean ± SEM, *n* = 4. ^$^ *p* < 0.01, * *p* < 0.05, compared to control.

Because the administered MK-4 dose (100 mg/kg BW) was relatively higher than usual pharmacological doses (45 mg/day for the treatment of osteoporosis) in humans, we investigated the effects of lower doses of MK-4 (10 or 50 mg/kg BW) on the mRNA levels of bile acid synthesis and energy homeostasis genes by qRT-PCR. Expression levels of *Cyp7a1* and *Cyp8b1* were also significantly suppressed by the treatment with lower doses of MK-4 in hPXR mice, but not in WT mice (Figure 5A). Energy homeostasis gene *Slc2a5* mRNA level was decreased by MK-4 at lower doses in hPXR mice (Figure 5B). However, in WT mice, *Pdk4* was significantly affected by the treatment with the lowest dose of MK-4 (Figure 5B).

### 3.6. Effects of MK-4 on mRNA Levels of Bile Acid Synthesis Genes in HepG2 Cells

**Figure 6:**
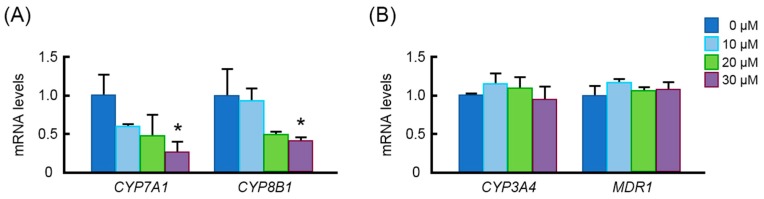
Effects of menaquinone-4 on gene expression in human hepatocarcinoma HepG2 cells. HepG2 cells were treated with menaquinone-4 for 24 h, and then mRNA levels of *CYP7A1* and *CYP8B1* (**A**) and *CYP3A4* and *MDR1* (**B**) were measured by quantitative RT-PCR. The relative mRNA levels were normalized to the amount of eukaryotic translation elongation factor 1α1 mRNA. Data are expressed as the mean ± SEM, *n* = 3. * *p* < 0.05, compared to control.

We sought to investigate the effects of MK-4 on the expression levels of *CYP7A1*, which encodes cholesterol 7α-hydroxylase, a rate limiting enzyme in bile acid synthesis, and of *CYP8B1*, which encodes sterol 12α-hydroxylase, another important enzyme in bile acid synthesis, in human hepatocarcinoma HepG2 cells. After 24 h of the treatment with 30 µM MK-4, the expression levels of both genes were markedly suppressed (Figure 6) even though mRNA levels of *CYP3A4* and *MDR1* were not changed.

## 4. Discussion

In the present study, we have shown that genes involved in drug metabolism as well as in bile acid synthesis and energy homeostasis were affected significantly by MK-4 treatment in hPXR mice. In WT mice, however, the effect of MK-4 on the expression of these genes was much weaker. Moreover, we also found that the MK-4 effect on gene expression was different from that of Rif.

It has been reported that MK-4 binds to and activates PXR, affecting bone homeostasis [14]. Moreover, the tumor-suppressive effect of MK-4 is also mediated by PXR [21]. However, the effect of VK on the genes involved in bile acid synthesis in hPXR mice has not been reported so far. In the present study, we used PXR humanized mouse line generated by replacing the mouse PXR LBD with the human PXR LBD. There are some advantages of this mouse line over other PXR humanized mice: the expression of PXR in different tissues is comparable to that in WT PXR and given that only LBD is replaced, the binding affinity of DBD of PXR to target genes in mice is not altered [4].

Masuyama et al. showed that PXR interacts with co-activators in ligand-species-dependent manner [29]. This characteristic of PXR indicates that different ligands are able to specifically change the conformation of PXR-LBD, resulting in different interactions with co-activators. This mechanism allows PXR to alter the expression of many genes involved in drug metabolism and homeostasis [29,30]. In our present study, we have also found that Rif and MK-4 specifically affected different sets of PXR target genes, even though both are able to bind to PXR.

PXR is a master regulator of drug metabolizing enzymes and transporters; it protects the body by facilitating the clearance of toxic substances. Therefore, initially, we have analyzed the effects of MK-4 on common PXR target genes, including some other genes involved in drug metabolism, and compared it with the effects of Rif on the expression of these genes in hPXR mice. *Ces2a*, *Cyp3a11*, *Gstp1*, and *Mdr1* are well-known PXR target genes [4,31]. We have found that expression levels of all these common PXR target genes were increased significantly by Rif treatment, as expected. However, although some genes (*Abca3*, *Cyp2s1*, and *Sult1b1*) encoding drug metabolizing proteins were affected by MK-4 treatment in hPXR mice, the expression levels of many common PXR target genes were not altered. Furthermore, we observed no significant effect of MK-4 treatment on the expression levels of typical PXR target genes in WT mice and rats (data not shown). These results indicate that MK-4 has little or no potency to modulate the expression levels of drug metabolism-related, endogenous PXR target genes in rodent liver.

*Abca3*, *Cyp2s1*, and *Sult1b1*, whose expression was modulated by MK-4, are not typical PXR target genes. From the in silico analysis of ChIP-Seq database, it was found that *Abca3* has a PXR binding site in its promoter region [32]. *Cyp2s1* is known to be regulated mainly by aryl hydrocarbon receptor (AHR). However, the regulation of *Cyp2s1* is species-dependent, and it was found that *Cyp2s1* is not regulated by the PXR ligand dexamethasone in male Sprague Dawley rats [33]. The contradictory finding in our study may be due to the difference in species used. *Cyp1a1* and *Sult1b1*, which also encode drug metabolizing proteins, are known AHR target genes regulated by PXR activation [31,34].

We sought to clarify whether any genes unrelated to drug metabolism were affected by MK-4 treatment in hPXR mice by DNA microarray assay. We found that some genes involved in bile homeostasis, including *Cyp7a1*, the gene encoding rate limiting enzyme of bile acid synthesis, were affected by MK-4 treatment in hPXR mice. Furthermore, some energy homeostasis genes were also affected. These findings were confirmed by qRT-PCR. To the best of our knowledge, these were the first observations of MK-4 treatment effects on the expression of bile acid synthesis genes.

Bile acids are physiological detergents required for the intestinal transport and absorption of lipids, including fat-soluble vitamins [35]. These amphipathic sterols are synthesized from cholesterol in the liver and secreted into the intestine where they function to emulsify dietary lipids. There are two pathways for the synthesis of bile acids: classic or neutral pathway and alternative or acidic pathway. The classic pathway is the major pathway, which is initiated by CYP7A1 enzyme. Cholesterol is converted into two primary bile acids in human liver—chenodeoxycholic acid (CDCA) and cholic acid (CA). The distribution of these two bile acids is determined by the activity of CYP8B1 enzyme. Most bile acids are conjugated to glycine or taurine to decrease their toxicity and increase solubility for the secretion into bile. In the intestine, glyco-and tauro-conjugated CA and CDCA are deconjugated and eventually transformed into secondary bile acids, deoxycholic acid (DCA), and lithocholic acid (LCA), respectively, by intestinal flora. CA, CDCA, DCA, and a small amount of LCA are transported back to the liver to inhibit bile acid synthesis [35].

Staudinger et al. have shown that PCN-mediated PXR activation regulates the expression of genes involved in the biosynthesis, transport, and metabolism of bile acids, including *Cyp7a1* in mice [10]. Hepatic nuclear factor 4α (HNF4α) and its coactivator, peroxisome proliferator-activated receptor γ coactivator α (PGC1α) are crucial transcription factors for the expression of *CYP7A1* and *CYP8B1*. It has been reported that liganded PXR interacts with HNF4α to inhibit the transcription of *CYP7A1* in human primary hepatocytes [36]*.* Another report suggested that Rif treatment seems to promote the dissociation of PGC1α from the promoters of *CYP7A1* and *CYP8B1* in HepG2 cells, because ligand-activated PXR interacts with PGC1α [37]. In our study, we have found that mRNA levels of *Cyp7a1* and *Cyp8b1* were significantly suppressed by MK-4 treatment in hPXR mice, but not in WT mice. We also found that MK-4 treatment for 24 h resulted in a significant suppression of both *CYP7A1* and *CYP8B1* mRNA in HepG2 cells. The biological significance of VK for the regulation of bile acids synthesis is not clear, but recently, Maldonado et al. reported that VK deficiency is associated with intrahepatic cholestasis of pregnancy in certain patients. Low levels of VK may cause dysregulation of bile acid synthesis with concomitant upregulation of *CYP7A1* and *CYP8B1* expression levels, which occurs during the progression of this disease [38].

Recent studies have revealed that PXR plays an important role in energy homeostasis by modulating the metabolism of lipids and glucose through direct gene regulation or through crosstalk with other transcriptional regulators. Being a complex regulator, PXR promotes lipogenesis but suppresses gluconeogenesis and β-oxidation of fatty acids [39,40]. Several groups of researchers have provided strong evidence that ligand-activated PXR works as an important regulator of glucose and lipid metabolism, thus affecting the overall energy homeostasis of the body [41]. However, there are also several contradictory outcomes regarding the significance of PXR in energy homeostasis because discrepant results have been obtained in mouse models. Variation in the results may be due to the use of different mouse models or methods that critically affected the results. Moreover, species-specific and even gender-specific differences in response to PXR activation may partly explain those variable results [41]. Notwithstanding these discrepancies, VK has been shown to have a beneficial effect on glucose homeostasis as well as on the sensitivity to insulin, although the mechanism of these actions has not been revealed so far [42].

Aldolase is a glycolytic enzyme that catalyzes the cleavage of fructose-1, 6-bisphosphate into dihydroxyacetone phosphate and glyceraldehyde-3-phosphate in the glycolytic pathway. In mammalian tissues, there are three primary forms of aldolase: aldolase A is found predominantly in the muscles, aldolase B—in the liver and kidney, and aldolase C—in the brain [43]. We did not find a significant effect of MK-4 on aldolase B mRNA level by DNA microarray analysis (data not shown). However, MK-4 treatment significantly upregulated the expression of *Aldoc* in WT mice, whereas in hPXR mice, *Aldoc* mRNA was significantly downregulated. Further research is necessary to explain this result.

Fructose absorption is mediated by the fructose transporter GLUT5 encoded by *Slc2a5* gene. *Slc2a5* is mainly expressed in the intestine [44]. A previous study revealed that ligand-mediated PXR activation induced significant hepatic *Slc2a5* expression in rats [45]. On the contrary, in the present study, we have found that *Slc2a5* expression was significantly suppressed by MK-4 treatment in hPXR mice.

These data indicate that MK-4 is likely a strong ligand of human PXR and a weak modulator of murine PXR in the regulation of metabolism-related genes. In addition, we showed that MK-4 affects expression levels of genes involved in bile synthesis and energy homeostasis likely through interaction with PXR in humans.

## 5. Conclusions

MK-4 affected expression levels of genes involved in drug metabolism, energy homeostasis and, importantly, bile acid synthesis possibly through the interaction with hPXR. Given that MK-4 is used as a nutraceutical, our observations suggest that there is a risk of undesired interactions between MK-4 and prescribed drugs. Therefore, awareness of this potential nutraceutical-drug interaction is critical. Furthermore, because MK-4 inhibits bile synthesis, MK-4 may have a protective effect in cholestasis.

## Figures and Tables

**Table 1:** Nucleotide sequences of primers used in quantitative RT-PCR.

Gene Symbol	Forward Primer	Reverse Primer
*Abca3*	TCCTTTGCCTTCATAGCACAGTT	GACCAAGCCAGGGATGCTAA
*Aldh1b1*	TCAGCCTGAGTTCCCAGTGA	GGTTGACACTTCTTGTAACACTTCA
*Aldoc*	TCATCTCACATCTCACATGCCTT	GGTCTCGGGTACAGCAATGT
*Ces2a*	GTATCGCTTGGGGGTCCTTG	GCCACTTGGTCCAGGTATCC
*Cyp1a1*	TGCATCAGCTCTTGGTCTCTC	CAAATGCATAAGCAAAATACAGTCCAT
*Cyp2s1*	AGCTGACCTCTTTGTGACTTGA	TTTATTGCAAAACGGGAACCTTCA
*Cyp39a1*	GAAGGTGGGGAACGGAAACT	CCTGGCACAAAACCAGATGC
*Cyp3a11*	CCTGGGTGCTCCTAGCAATC	GGCCCAGGAATTCCCTGTTT
*Cyp7a1*	AAAACAAGTTTTATGACTCCCTGAAC	GGAAGGTATGTGGATACATTCAGTT
*Cyp8b1*	AAACCTGGAAAAAGACGGCATAAG	ACTGAAGCATGTAGCCTAACCAA
*Eef1al*	GATGGCCCCAAATTCTTGAAG	GGACCATGTCAATGGCAG
*Gstp1*	CAGGGCCAGAGCTGGAAG	AGCCTTGCATCCAGGTATCTATG
*Lipin1*	TGGGTACCATACATTTCAAAGTTGC	CTATGGAGCAAGTCGCTCATTTC
*Mdr1*	ATGCTGAGACAGGATGTGAGC	AGACCCTGTAGCCCCTTTCA
*Nceh1*	TTTTTCATATGTGTCCATGTCTGGG	ACCAGTACTCATATGCACATACCC
*Pdk4*	ATGAACCCATGGGAGACTTTAA	GCCTGGGCATTTAGCATCTAT
*Slc2a5*	CCATTTGCGAAGACACACTGAG	CGCTTACAGTTAATAATCCACGCTC
*Sult1b1*	GACCCACAATCTGATACAACCTC	AAATTAGTCTAGGTCACAGATGCTT
*hCYP3A4*	TGGTGATGATTCCAAGCTATGCT	AATGCAGTTTCTGGGTCCACTTC
*hCYP7A1* *	AAACGGGTGAACCACCTCTAGA	AACTCAAGAGGATTGGCACCA
*hCYP8B1*	AAACGGGTGAACCACCTCTAGA	AACTCAAGAGGATTGGCACCA
*hMDR1*	CCCATCATTGCAATAGCAGG	TGTTCAAACTTCTGCTCCTGA

* Mitro et al. [28].

**Table 2:** Body and liver weights of wild type and hPXR mice treated by menaquinone-4.

Groups		Body Weight (g)	Liver Weight (g)	Ratio (Liver/Body Weight)
Wild type	Control	19.7 ± 0.440 *	0.740 ± 0.0386	0.0375 ± 0.00160
	10 mg/kg BW	20.1 ± 0.614	0.7875± 0.0175	0.0392 ± 0.00072
	50 mg/kg BW	20.6 ± 0.795	0.770 ± 0.0409	0.0377 ± 0.00169
	100 mg/kg BW	19.4 ± 0.290	0.807 ± 0.0169	0.0417 ± 0.00030
hPXR	Control	18.0 ± 0.422	0.776 ± 0.0281	0.0436 ± 0.0025
	10 mg/kg BW	17.3 ± 0.414	0.798 ± 0.0253	0.0461 ± 0.00081
	50 mg/kg BW	17.4 ± 0.232	0.808 ± 0.0317	0.0465 ± 0.00218
	100 mg/kg BW	18.7 ± 0.396	0.822 ± 0.0348	0.0439 ± 0.00091

* Data are expressed as the mean ± SEM, *n* = 4–9.

**Table 3:** Comparison of fold changes of gene expression levels (compared to corresponding control) in wild-type and hPXR mice.

Function	Gene Symbol	WT MK-4 *	hPXR MK-4	hPXR Rif
Bile homeostasis	*Cyp7a1*	2.24	0.07	0.39
	*Cyp8b1*	1.11	0.45	0.99
	*Cyp39a1*	0.93	1.82	1.12
	*Nceh1*	0.97	1.83	1.91
Energy homeostasis	*Aldoc*	1.44	0.44	0.73
	*Lipin1*	1.03	2.00	0.75
	*Pdk4*	0.61	2.12	0.96
	*Slc2a5*	0.99	0.51	0.71

* WT, wild type; MK-4, menaquinone-4; Rif, rifampicin.

## References

[1] Kliewer S.A., Moore J.T., Wade L., Staudinger J.L., Watson M.A., Jones S.A., McKee D.D., Oliver B.B., Willson T.M., Zetterstrom R.H. (1998). An Orphan Nuclear Receptor Activated by Pregnanes Defines a Novel Steroid Signaling Pathway. Cell.

[2] Bertilsson G., Heidrich J., Svensson K., Asman M., Jendeberg L., Sydow-Backman M., Ohlsson R., Postlind H., Blomquist P., Berkenstam A. (1998). Identification of a human nuclear receptor defines a new signaling pathway for CYP3A induction. Proc. Natl. Acad. Sci. USA.

[3] Blumberg B., Sabbagh W., Juguilon H., Bolado J., van Meter C.M., Ong E.S., Evans R.M. (1998). SXR, a novel steroid and xenobiotic-sensing nuclear receptor. Genes Dev..

[4] Igarashi K., Kitajima S., Aisaki K., Tanemura K., Taquahashi Y., Moriyama N., Ikeno E., Matsuda N., Saga Y., Blumberg B. (2012). Development of humanized steroid and xenobiotic receptor mouse by homologous knock-in of the human steroid and xenobiotic receptor ligand binding domain sequence. J. Toxicol. Sci..

[5] Guengerich F.P. (1999). Cytochrome P-450 3A4: Regulation and role in drug metabolism. Ann. Rev. Pharmacol. Toxicol..

[6] Watkins R.E., Davis-Searles P.R., Lambert M.H., Redinbo M.R. (2003). Coactivator Binding Promotes the Specific Interaction Between Ligand and the Pregnane X Receptor. J. Mol. Biol..

[7] Watkins R.E., Wisely G.B., Moore L.B., Collins J.L., Lambert M.H., Williams S.P., Willson T.M., Kliewer S.A., Redinbo M.R. (2001). The Human Nuclear Xenobiotic Receptor PXR: Structural Determinants of Directed Promiscuity. Science.

[8] Watkins R.E., Maglich J.M., Moore L.B., Wisely G.B., Noble S.M., Davis-Searles P.R., Lambert M.H., Kliewer S.A., Redinbo M.R. (2003). 2.1 Å Crystal Structure of Human PXR in Complex with the St. John’s Wort Compound Hyperforin. Biochemistry.

[9] Zhou C., Verma S., Blumberg B. (2009). The steroid and xenobiotic receptor (SXR), beyond xenobiotic metabolism. Nucl. Recept. Signal..

[10] Staudinger J.L., Goodwin B., Jones S.A., Hawkins-Brown D., MacKenzie K.I., LaTour A., Liu Y., Klaassen C.D., Brown K.K., Reinhard J. (2001). The nuclear receptor PXR is a lithocholic acid sensor that protects against liver toxicity. Proc. Natl. Acad. Sci. USA.

[11] Ohsaki Y., Shirakawa H., Miura A., Giriwono P.E., Sato S., Ohashi A., Iribe M., Goto T., Komai M. (2010). Vitamin K suppresses the lipopolysaccharide-induced expression of inflammatory cytokines in cultured macrophage-like cells via the inhibition of the activation of nuclear factor κB through the repression of IKKα/β phosphorylation. J. Nutr. Biochem..

[12] Ito A., Shirakawa H., Takumi N., Minegishi Y., Ohashi A., Howlader Z.H., Ohsaki Y., Sato T., Goto T., Komai M. (2011). Menaquinone-4 enhances testosterone production in rats and testis-derived tumor cells. Lipids Health Dis..

[13] Lamson D.W., Plaza S.M. (2003). The Anticancer Effects of Vitamin K. Altern. Med. Rev..

[14] Tabb M.M., Sun A., Zhou C., Grun F., Errandi J., Romero K., Pham H., Inoue S., Mallick S., Lin M. (2003). Vitamin K2 Regulation of Bone Homeostasis Is Mediated by the Steroid and Xenobiotic Receptor SXR. J. Biol. Chem..

[15] Beulens J.W., van der A D.L., Grobbee D.E., Sluijs I., Spijkerman A.M., van der Schouw Y.T. (2010). Dietary Phylloquinone and Menaquinones Intakes and Risk of Type 2 Diabetes. Diabetes Care.

[16] Neogi T., Booth S.L., Zhang Y.Q., Jacques P.F., Terkeltaub R., Aliabadi P., Felson D.T. (2006). Low Vitamin K Status Is Associated With Osteoarthritis in the Hand and Knee. Arthritis Rheum..

[17] Presse N., Shatenstein B., Kergoat M.J., Ferland G. (2008). Low Vitamin K Intakes in Community-Dwelling Elders at an Early Stage of Alzheimer’s Disease. J. Am. Diet. Assoc..

[18] Geleijnse J.M., Vermeer C., Grobbee D.E., Schurgers L.J., Knapen M.H., van der Meer I.M., Hofman A., Witteman J.C. (2004). Dietary Intake of Menaquinone Is Associated with a Reduced Risk of Coronary Heart Disease: The Rotterdam Study. J. Nutr..

[19] Azuma K., Inoue S. (2017). Vitamin K, SXR, and GGCX. Vitamin K_2_—Vital for Health and Wellbeing.

[20] Ichikawa T., Horie-Inoue K., Ikeda K., Blumberg B., Inoue S. (2006). Steroid and Xenobiotic Receptor SXR Mediates Vitamin K2-activated Transcription of Extracellular Matrix-related Genes and Collagen Accumulation in Osteoblastic Cells. J. Biol. Chem..

[21] Azuma K., Urano T., Ouchi Y., Inoue S. (2009). Vitamin K2 Suppresses Proliferation and Motility of Hepatocellular Carcinoma Cells by Activating Steroid and Xenobiotic Receptor. Endocr. J..

[22] Jones S.A., Moore L.B., Shenk J.L., Wisely G.B., Hamilton G.A., McKee D.D., Tomkinson N.C., LeCluyse E.L., Lambert M.H., Willson T.M. (2000). The Pregnane X Receptor: A Promiscuous Xenobiotic Receptor That Has Diverged during evolution. Mol. Endocrinol..

[23] Xie W., Barwick J.L., Downes M., Blumberg B., Simon C.M., Nelson M.C., Neuschwander-Tetri B.A., Brunt E.M., Guzelian P.S., Evans R.M. (2000). Humanized xenobiotic response in mice expressing nuclear receptor SXR. Nature.

[24] Ma X., Shah Y., Cheung C., Guo G.L., Feigenbaum L., Krausz K.W., Idle J.R., Gonzalez F.J. (2007). The Pregnane X Receptor Gene-Humanized mouse: A Model for Investigating drug-drug Interactions Mediated by Cytochromes P450 3A. Drug Metab. Dispos..

[25] Scheer N., Ross J., Rode A., Zevnik B., Niehaves S., Faust N., Wolf C.R. (2008). A novel panel of mouse models to evaluate the role of human pregnane X receptor and constitutive androstane receptor in drug response. J. Clin. Investig..

[26] Ohsaki Y., Shirakawa H., Hiwatashi K., Furukawa Y., Mizutani T., Komai M. (2006). Vitamin K Suppresses Lipopolysaccharide Induced Inflammation in the Rat. Biosci. Biotechnol. Biochem..

[27] Sato S., Shirakawa H., Tomita S., Tohkin M., Gonzalez F.J., Komai M. (2013). The aryl hydrocarbon receptor and glucocorticoid receptor interact to activate human metallothionein 2A. Toxicol. Appl. Pharmacol..

[28] Mitro N., Godio C., De Fabiani E., Scotti E., Galmozzi A., Gilardi F., Caruso D., Chacon A.B.V., Crestani M. (2007). Insights in the Regulation of Cholesterol 7α-Hydroxylase Gene Reveal a Target for Modulating Bile Acid Synthesis. Hepatology.

[29] Masuyama H., Hiramatsu Y., Kunitomi M., Kudo T., MacDonald P.N. (2000). Endocrine Disrupting Chemicals, Phthalic Acid and Nonylphenol, Activate Pregnane X Receptor-Mediated Transcription. Mol. Endocrinol..

[30] Pavek P. (2016). Pregnane X Receptor (PXR)-Mediated Gene Repression and Cross-Talk of PXR with Other Nuclear Receptors via Coactivator Interactions. Front. Pharmacol..

[31] Tolson A.H., Wang H. (2010). Regulation of drug-metabolizing enzymes by xenobiotic receptors: PXR and CAR. Adv. Drug Deliv. Rev..

[32] Benson E.A., Eadon M.T., Desta Z., Liu Y., Lin H., Burgess K.S., Segar M.W., Gaedigk A., Skaar T.C. (2016). Rifampin Regulation of Drug Transporters Gene Expression and the Association of MicroRNAs in Human Hepatocytes. Front. Pharmacol..

[33] Wang B., Robertson L.W., Wang K., Ludewig G. (2011). Species difference in the regulation of cytochrome P450 2S1: Lack of induction in rats by the aryl hydrocarbon receptor agonist PCB126. Xenobiotica.

[34] Maglich J.M., Stoltz C.M., Goodwin B., Hawkins-Brown D., Moore J.T., Kliewer S.A. (2002). Nuclear Pregnane X Receptor and Constitutive Androstane Receptor Regulate Overlapping but Distinct Sets of Genes Involved in Xenobiotic Detoxification. Mol. Pharmacol..

[35] Chiang J.Y. (2009). Bile acids: Regulation of synthesis. J. Lipid Res..

[36] Li T., Chiang J.Y. (2005). Mechanism of rifampicin and pregnane X receptor inhibition of human cholesterol 7α-hydroxylase gene transcription. Am. J. Physiol. Gastrointest. Liver Physiol..

[37] Bhalla S., Ozalp C., Fang S., Xiang L., Kemper J.K. (2004). Ligand-activated Pregnane X Receptor Interferes with HNF-4 Signaling by Targeting a Common Coactivator PGC-1α. Functional implications in hepatic cholesterol and glucose metabolism. J. Biol. Chem..

[38] Maldonado M., Alhousseini A., Awadalla M., Idler J., Welch R., Puder K., Patwardhan M., Gonik B. (2017). Intrahepatic Cholestasis of Pregnancy Leading to Severe Vitamin K Deficiency and Coagulopathy. Case Rep. Obstet. Gynecol..

[39] Wada T., Gao J., Xie W. (2009). PXR and CAR in energy metabolism. Trends Endocrinol. Metab..

[40] Zhou J., Zhai Y., Mu Y., Gong H., Uppal H., Toma D., Ren S., Evans R.M., Xie W. (2006). A Novel Pregnane X Receptor-mediated and Sterol Regulatory Element-binding Protein-independent Lipogenic Pathway. J. Biol. Chem..

[41] Hakkola J., Rysa J., Hukkanen J. (2016). Regulation of hepatic energy metabolism by the nuclear receptor PXR. Biochim. Biophys. Acta.

[42] Prasenjit Manna P., Kalita J. (2016). Beneficial role of vitamin K supplementation on insulin sensitivity, glucose metabolism, and the reduced risk of type 2 diabetes: A review. Nutrition.

[43] Rutter W.J. (1964). Evolution of Aldolase. Fed. Proc..

[44] Burant C.F., Takeda J., Brot-Laroche E., Bell G.I., Davidson N.O. (1992). Fructose Transporter in Human Spermatozoa and Small Intestine is GLUT5. J. Biol. Chem..

[45] Hartley D.P., Dai X., He Y.D., Carlini E.J., Wang B., Huskey S.W., Ulrich R.G., Rushmore T.H., Evers R., Evans D.C. (2004). Activators of the Rat Pregnane X Receptor Differentially Modulate Hepatic and Intestinal Gene Expression. Mol. Pharmacol..

